# Magnetic Composites for Advanced Characterization of Magnetic Field Sensors and Biosensors

**DOI:** 10.3390/s26123794

**Published:** 2026-06-14

**Authors:** Ekaterina A. Burban, Alexander P. Safronov, Ksenia O. Il’inova, Grigory Yu. Melnikov, Andrey V. Svalov, Igor V. Beketov, Anton A. Yushkov, Galina V. Kurlyandskaya

**Affiliations:** 1 Institute of Natural Sciences and Mathematics, Ural Federal University, 19 Mira Str., Ekaterinburg 620002, Russia; 2 Institute of Electrophysics, UB, RAS, 106 Amundsena Str., Ekaterinburg 620016, Russia

**Keywords:** iron nanoparticles, gadolinium rapidly quenched ribbons, iron oxide nanoparticles, mechanosynthesis, filled magnetic composites, FeNi/Cu multilayered structures, stray fields, magnetic field sensors, magnetic biosensors, magnetoimpedance

## Abstract

Gadolinium is a rare-earth element that is promising for the field of biomedicine due to its unique properties that enhance image quality, giving it high potential in targeted cancer therapy, antimicrobial treatments, etc. The disadvantage of Gd-containing materials is their high toxicity. In this work, ensembles of Fe and Al_2_O_3_ nanoparticles were fabricated by the electric explosion of wire and Gd ribbons using rapid quenching techniques. Stable Fe, Fe/Gd and Fe/Gd/Al_2_O_3_ aqueous suspensions with a Z-potential of about −54 mV were fabricated by the ball-milling mechanosynthesis of Fe (100%), Fe and Gd (70 and 30 wt. % accordingly) and Fe, Al_2_O_3,_ and Gd (69, 30 and 1 wt.% accordingly). Fillers from suspensions were used for the synthesis of epoxy composites mimicking natural tissue with embedded magnetic particles. The concentration range for synthesized epoxy composites (0, 5, 10, and 15 wt.% of the filler) corresponded to the biomedical range of interest. Thin-film magnetoimpedance (MI) elements were prepared by a sputtering technique: conventional [FeNi/Cu]_5_/Cu/[Cu/FeNi]_5_ (NP) element and [FeNi/Cu]_5_/Cu/[Cu/P{FeNi]_5_} element with patterned top multilayer (SqP). They showed a maximum MI ratio of about 160% for NP and about 60% for SqP. MI sensor response was affected by the presence of filled magnetic composites in the shape of cylinders (5 mm × 4 mm) situated at about 1 mm due to the stray fields in the filler. MI response showed a linear dependence on the filler concentration for each selected position. These results open the possibility to develop new iron- and gadolinium-containing materials for simultaneous magnetic imaging and detection by magnetic field sensors, extending the functional properties of Fe/Gd materials for biomedical devices and therapies.

## 1. Introduction

Magnetic nanoparticles (MNPs) have been the subject of intensive research over the last 50–60 years [1,2]. They represent the family of magnetic materials in which small dimensions result in the appearance of new functional properties [3,4]. The emergence of microelectronic, information, aerospace and automobile technologies became possible in many ways due to the development of magnetic MNPs and composites fabricated on their basis [5]. In addition, many of our expectations regarding the further advances in modern technologies are precisely connected with this class [6,7,8]. However, the greatest interest of the scientific community and practitioners has been drawn to magnetic nanoparticles that focus on biomedical applications (bioimaging, drug delivery, theragnostics, magnetic biosensing, etc.) [9,10,11]. Biomagnetic signals are very small [11,12,13,14] and, therefore, even barely noticeable external disturbances affecting the accuracy of measured parameters or therapeutic parameters can be critical for achieving the desired result [15,16,17,18].

A magnetic biosensor is a compact analytical device, incorporating a biological-substance material, associated with a physicochemical magnetic transducer, which has the capability to define the changes in magnetic field strength [16]. Magnetic sensors for biomedical research can be divided into three main types, based on the goal of application: analysis of electric and/or magnetic properties of biosystems connected with their functional state, analysis of the desired properties of the biological analytes, and analysis of the desired properties of the biological tissue or whole organism. The magnetic biosensors, as diagnostic tools, related to the third case possess the highest level of demand in terms of device functionality, due to their high sensitivity to the external magnetic field, at a level close to pico-Tesla [13].

An analysis of electric and magnetic properties of biosystems connected with their functional state is typically related to the analysis of self-induced changes in the properties of living systems, and this does not require magnetic label injection. However, an analysis of select properties of the biological analytes and an analysis of the desired properties of the biological tissue or whole organism can be made by magnetic biosensors based on the principle of magnetic label detection, i.e., the detection of the sum of stray fields of magnetizable nanoparticles working as magnetic markers.

**Figure 1:**
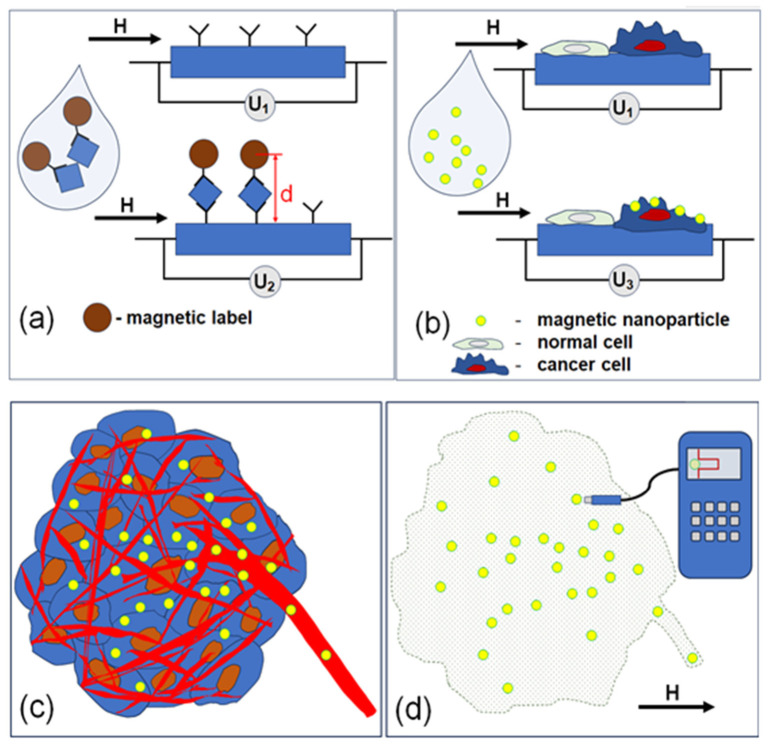
Magnetic label detection: (**a**)—description of the working principle for analysis of the biological analytes using magnetic labels, causing the change in the magnetic field sensor output from U_1_ to U_2_; d is the distance between the center of the magnetic label and the sensor element, H is an external magnetic field; (**b**)—working principle for analysis of the number of magnetic nanoparticles internalized by cancer cells, causing the change in the initial value of the sensor output U_1_ to U_3_; (**c**)—scheme of the tumor with developed blood vessels (red color) transporting magnetic nanoparticles (yellow color); (**d**)—the magnetic stray fields of MNPs distributed over the tumor can be measured by magnetic field detector.

Figure 1 describes the main points of the three most-requested configurations for magnetic label detection. Figure 1a is the case for the properties of biological analytes. The surface of a magnetic field sensor is functionalized with a specific antibody, and the output signal is given as the initial voltage drop U_1_. The sample to analyze is prepared on the surface of the magnetic-sensitive element in such a way that magnetic labels with their surface covered by a specific antigen form complexes with the biocomponent of interest: one biocomponent is associated with one magnetic label. An analyte with such complexes is placed onto the surface of the magnetic-sensitive element, causing the formation of new complexes (antibody–magnetic label–antigen), ensuring the position of all of the magnetic labels at a distance d from the surface of the magnetic-sensitive element. As a result, the response in the sensitive element changes from U_1_ to U_2_. In the case of proper calibration, there is a simple dependence |U_1_ − U_2_| = kN, where N is the number of magnetic labels, equal to the number of biocomponents, and k is a coefficient defined during calibration.

Figure 1a describes the case of magnetic labels that are situated close to the surface of the sensitive elements at distances comparable to the size of the antigen or antibody molecules. The diameter of the magnetic labels, typically magnetic nanoparticles, is close to a few nanometers. In this case, the contribution of the stray fields of MNPs can be detected by magnetic sensors working on different principles [10,13,14,16]. For practical purposes, there is a need to measure the number of MNPs internalized by living cells. Figure 1b shows the case for the mixture of normal and cancer cells carrying different numbers of MNPs due to their specific uptake. That is, MNPs are able to pass through the cell membranes of the cancer cells, and they are not internalized by the normal cells.

It is important to control the number of MNPs in the cancer cells. It this case, MNPs are situated at different distances from the magnetic-sensitive element, and our understanding of the connection |U_1_ − U_3_| = f(N) is much more complex. The distances in the second case become on the order of the size of the living cells (on the order of 100 μm). Such an increase in the distances dramatically increases the demand to the magnetic field detector sensitivity, as the intensity of the stray fields shows 1/d^3^ decay with distance. One of the most promising types of magnetic effects ensuring very high sensitivity with respect to external magnetic field, magnetoimpedance (MI), has already demonstrated the capability of such an evaluation [11,12,13,14]. Even so, there is a practical request that is even more demanding: the evaluation of the concentration of MNPs in living tissues, where specific distances between each MNP and the sensitive element are different, and their average value can be on the order of centimeters (Figure 1c,d). The situation for the detection of the number of MNPs in cancer tissues is even more complex, as the angiogenesis (the physiological process for new blood vessel formation which, while vital for health, also enables accelerated tumor growth by feeding cancer cells) of the tumor results in a very complex structure of cancer-affected tissues [17]. Magnetic nanoparticles therefore do not become randomly distributed throughout the tumor but, to some extent, their distribution reflects the structure of the cancer tissue.

Scanning the surface of the body using a magnetic field sensor may help to create a reasonable map of the tumor position if the tumor incorporates MNPs. In this case, one should ensure that the scanning material is a polymer composite filled with magnetic nanoparticles. For the accurate calibration of the measuring device for the analysis of select properties of the biological analytes and the analysis of the magnetic properties of tumor tissues, different calibration protocols and calibration samples can be appropriate. This indicates the need to elaborate calibration protocols and calibration samples, which are the synergetic combination of two different magnetic materials that successfully work in one device; the need for the advanced characterization of the filled polymer composites has developed as have calibration samples.

Recently, rare-earth (RE) elements in biomedical applications have been intensively investigated, showing that doping biomaterials with RE elements can enhance their functional properties. Gadolinium is a biocompatible element that is most promising in the biomedical field. Because of its unique paramagnetic properties, it enhances image quality. Emerging research is also exploring its potential in targeted cancer therapy, antimicrobial treatments, bone regeneration, and bioprobes. The disadvantage of Gd-based materials is its high toxicity level. The concept of gadolinium toxicity refers to adverse reactions caused by the retention of Gd-based contrast agents used in biomedical applications for quality imaging. Free gadolinium is highly toxic to human cells and, therefore, at present, it is administered in a bound form. Even so, these pharmacological agents can dissociate, resulting in the formation of free metal deposits in tissues and in severe health damage [18]. Recently, Gd’s potential applicability has also been addressed with respect to the field of theranostic applications, combining the advantages of both therapy and diagnostics [19]. At the same time, due to its paramagnetic state in biological conditions, it cannot be used for field-assisted drug delivery. Therefore, research is underway to create gadolinium-containing iron-based materials. In this research area, the need for transition from metallic iron to its bound form has long been recognized by researchers. The challenge is to create materials that would be slightly less sensitive to the replacement of some gadolinium with iron (even in bound forms), but would be able to serve new functions such as in therapy, targeted drug delivery, and magnetic detection. Iron oxide-based materials demonstrate much poorer image quality, but they can be used for multifunctional devices. For example, the combination of iron and gadolinium in one pharmacological product could reduce the side effects by the in-situ control of the concentration of the contrast agent by using weak magnetic field detectors [20].

In this work, large batches of iron- and gadolinium-based magnetic materials were fabricated by mechanosynthesis in stable aqueous solutions, followed by the fabrication of filled magnetic epoxy composites (0, 5, 10, and 15 wt.% of the filler extracted from the suspension) in the concentration range corresponding to the range of biomedical interest. Epoxy composite samples were carefully characterized and used for the calibration of thin-film magnetoimpedance elements for the non-contact definition of the concentration of MNPs in each composite, ensuring the possibility of the successful detection of proposed multilayer structures and synthesized iron plus gadolinium materials for multifunctional purposes, including magnetic biosensing.

## 2. Materials and Methods

Magnetic nanoparticles of iron (α-Fe) and iron oxides such as magnetite (Fe_3_O_4_) or maghemite (γ-Fe_2_O_3_) have potential interest for biomedical and technological applications. α-Fe is toxic and requires special effort for its careful encapsulation and isolation from the living system. However, keeping in mind that the saturation magnetization of α-Fe in the bulk state is more than two times higher than the saturation magnetization of magnetite or maghemite, these difficulties are worth overcoming. Both magnetite and maghemite usually demonstrate very high biocompatibility and, while falling short of pure iron in terms of saturation magnetization, they can compete with iron in bioapplications while also having other advantages [9]. Contrast agents are defined as agents capable of increasing the clarity of diagnostic images. Superparamagnetic iron oxide-containing magnetic resonance contrast agents are composites of iron oxide MNPs coated by pharmaceuticals like dextran or carboxydextran [17]. However, despite the lack of biocompatibility, for imaging cases and potentially for hyperthermia in bioapplications, Gd-based materials attract special attention [20,21]. Here, we propose to attempt to combine both materials and obtain both Fe- and Gd-based polymer composites, aiming to examine the possibility to control their concentration via a non-contact way using a magnetic field detector.

MNPs of iron were obtained by an electrophysical technique: the electric explosion of wire (EEW), providing spherical MNPs at production rates of up to 400 g/h in a single batch [22]. EEW includes the overheating and evaporation of a metallic wire (here ST-3, MS-group, Moscow, Russia) using an electric pulse at high power in a controlled atmosphere. More details about the conditions of fabrication and physicochemical properties of the same batch can be found elsewhere [22]. In addition, MNPs of Al_2_O_3_ were also fabricated by the EEW technique under the following conditions: wire diameter (A7E, Energo Servis, Ekaterinburg, Russia)—0.8 mm, wire length—150 mm, charging voltage—32 kV, capacitor bank capacity—4.8 μF, explosion frequency—0.7 Hz, gas environment—argon–oxygen mixture, oxygen concentration—30% by volume, pressure—1.1 bar, resulting wire superheat—0.75. Aluminum oxide Al_2_O_3_ (alumina) is a ceramic material characterized by its high hardness and very high melting point, providing excellent resistance to wear or corrosion. In this work, it was fabricated as a small addition during the ball-milling synthesis of one of the magnetic fillers. It was expected to play the role of an abrasive.

Gd rapidly quenched ribbons were obtained from pure gadolinium (S-target, Goodfellow, Hamburg, Germany). As the first step in the fabrication of Fe/Gd magnetic filler, rapidly quenched gadolinium ribbons (3 mm wide and 70 μm thick) were obtained using a previously elaborated protocol [23]. Afterwards, they were mechanically cut into small pieces a few millimeters long. Three types of the samples were prepared for ball-milling processing: (1) α-Fe MNPs—100% (sample denominated as Fe); (2) α-Fe MNPs—70 wt. % + Gd ribbon—30 wt. % (sample denominated as Fe/Gd); (3) α-Fe MNPs—69 wt. % + Gd ribbon—30 wt. % + Al_2_O_3_ MNPs (sample denominated as Fe/Gd/Al_2_O_3_).

The ball-milling technique was implemented using a mixer/mill device consisting of a hardened steel chamber filled with the balls and primary materials to mill. The number of balls was chosen such that the mass of the material had a ball-to-powder weight ratio of 66:1. The milling process was performed in ethylic alcohol for 24 h, followed by partial drying to a paste-like state and additional processing in a ball-mill in an aqueous solution of 0.2% sodium citrate for 10 h. Sodium citrate (Na_3_C_6_H_5_O_7_, SC, AMK-Group, Ekaterinburg, Russia) is a well-known electrostatic stabilizer [24]. Its addition results in a double electrical layer formation as follows. The surface of the nanoparticles adsorbs the negatively charged citrate ions, becoming negatively electrically charged. The adsorbed citrate ions attract positively charged sodium ions from the water solution due to electrostatic interactions. As a result of such treatment, two fractions were formed: stable suspension of MNPs and sediment. The stable suspension of MNPs was dried, and corresponding MNPs were used for the physicochemical characterization and synthesis of filled composites. For completeness, the same ball-milling device and the same experimental steps were used for the fabrication of magnetic fillers starting with Fe MNPs and Fe/Gd/A_2_O_3_ mixtures in the concentrations described above.

Physicochemical properties of magnetic fillers were studied using standard techniques. The evaluation of the phase composition and average size of the coherent diffraction domain were completed by an X-ray diffraction (XRD) method, implemented with a standard Bruker D8 Discover diffractometer (Bruker Corporation, Billerica, MA, USA) operating with Cu-K_α_ radiation (the wavelength λ = 1.5418 Å). Data analysis was carried out using TOPAS-3.0 software (Bruker, Moscow, Russia). The average size of coherent diffraction domains was estimated by the Scherrer equation. Transmission electron microscopy (TEM) was performed using a JEOL JEM2100 microscope (JEOL Corp., Tokyo, Japan) operated at 200 kV. In addition, energy-dispersive analysis (EDX) was realized for the MI element structural evaluation (see description below). The specific surface area of each MNP batch was measured by the Brunauer–Emmett–Teller (BET) low-temperature nitrogen adsorption technique with a Micromeritics TriStar3000 automatic sorption analyzer (Micromeritics, Norcross, GA, USA). Dynamic light scattering (DLS) was used to determine the size distribution profile of MNPs in suspensions after mechanosynthesis.

The magnetic properties of MNPs and composites were measured by the Lake Shore Cryotronics vibrating sample magnetometer (VSM) (Lake Shore 7404, Westerville, OH, USA).

To prepare samples for differential scanning calorimetry (DSC) and MI testing, first, MNPs in 0, 5, 10, and 15% content in epoxy resin were mechanically homogenized with ED-20. Then, TETA hardener was added in 1:6 proportion to ED-20. The curing process was studied using a DSC-131 instrument (SETARAM, Caluire, Auvergne, France) in linear polythermal mode, starting from −10 °C up to 250 °C, at heating rates of 2, 5, 10 K/min. For MI measurements, magnetic composites were synthesized in the shape of cylinders with a diameter of 5.0 mm and a height of 4.0 mm.

We are developing a laboratory prototype of magnetic field sensors and calibration samples (to work together), which can be useful for magnetic label detection. For tumor visualization and therapy, there is a problem with the detection of the malignant tissue at the early period of tumor development (tumor below 10 mm). A spherical shape could be biologically better for calibration samples, but it is much more difficult to fabricate composites with uniform structure in the shape of a sphere. In our case, the synthesis was done in a polymer tube up to 5 cm in length, which was further cut into smaller cylinders. We estimated the combination of 10.0 mm × 0.5 mm dimensions for the MI element and 5.0 mm × 40 mm dimensions for the calibration sample as optimum combination.

Magnetically soft Fe19Ni81 thin films are a well-known choice for different electronic applications (inductors, transformers and magnetic sensors) because, close to 80 percent nickel, the saturation magnetostriction constant for an alloy of this composition goes through zero and the material has the highest magnetic permeability and small coercivity, which provides an opportunity to induce a well-defined magnetic anisotropy with narrow local anisotropy axes distribution [25]. Therefore, Fe_19_Ni_81_ thin-film components were selected for the design of the magnetoimpedance-sensitive element. Previously, the scientific community has made special efforts to define the optimum parameters for obtaining the highest properties of magnetoimpedance multilayers consisting of two magnetic layers separated by a central conductive lead [26]. For Fe_19_Ni_81_, the thickness, number of layers, and material of the spacers were studied and discussed in detail [27].

It was shown in previous MI studies of different groups that the highest MI effect (in general) can be obtained in the three-layered sandwich structure ferromagnet–conductor–ferromagnet with similar thicknesses of magnetic layers and a conductive central part [16]. Therefore, the total thickness of the top and bottom magnetic layers in the present study was the same, and the distances were selected to be close to the theoretically calculated optimum and technologically possible for the deposition using the metallic masks.

An FeNi-based multilayered structure with patterned top multilayer is expected to provide benefits in accordance with the theoretical prediction made by the Buznikov et al. [28]. An electrodynamic model has been developed, allowing one to find the transverse permeabilities for the top (patterned) and bottom (non-patterned) layers of the MI structure by the standard procedure of solving the linearized Landau–Lifshitz equation and the equation for equilibrium magnetization angles. In the case of a profiled structure, a decrease in the angle of the deviation of the effective magnetic anisotropy axis from the transverse direction results in an increase in the permeability of the top layer, leading to the enhancement of the skin and magnetoimpedance effects [28].

**Figure 2:**
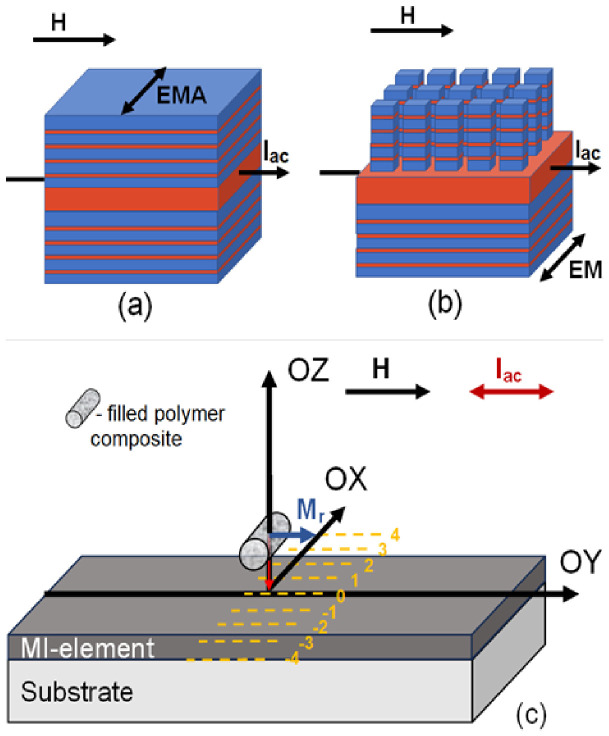
General scheme of the structure of two MI elements—(**a**) conventional multilayered structure (non-patterned, NP) and (**b**)—multilayered structure with patterned top multilayer (SqP—square patterned); schematic description of the measurements of filled polymer composite sample properties using MI thin-film element: H—external applied magnetic field, I_ac_—driving current intensity (both oriented parallel to the long side of the MI element), M_r_—remnant magnetization of the composite. Numbers from −4 to 4 correspond to displacements in millimeters (for MI measurements with composites), with 0 being a position at which the projection of the center of composite is situated in a central point of MI element (**c**).

Thin-film MI structures (Figure 2) were obtained by magnetron sputtering (Orion 8, AJA International Inc., Hingham, MA, USA) in radio frequency mode. Samples were deposited in an argon atmosphere with a gas purity of 99.98%. Deposition parameters were selected as follows: base pressure of 1.3 × 10^−6^ mbar, working argon pressure in the chamber of 3.9 × 10^−3^ mbar, and target voltage of 1.5 kV. Fe_19_Ni_81_ permalloy was selected as the composition showing the best magnetic properties.

There were two different types of te MI-sensitive elements (Figure 2): conventional [FeNi(100 nm)/Cu(3 nm)]_5_/Cu(500 nm)/[Cu(3 nm)/FeNi(100 nm)]_5_ with symmetric geometry of top and bottom (NP-non-patterned), and asymmetric [FeNi(100 nm)/Cu(3 nm)]_5_/Cu(500 nm)/[Cu(3 nm)/P{FeNi(100 nm)]_5_} element with patterned top multilayered structure (SqP—square-patterned). The patterning of the top multilayer was provided during the deposition process through Cu-mesh fabricated for electron microscopy purposes. As a result, parallel rows of squares of about 0.08 mm × 0.08 mm were formed, with the edges parallel to the main sides of the rectangular MI element.

The general elongated rectangular shape of the element was formed due to deposition through the rectangular metallic masks, ensuring 10.0 mm × 0.5 mm dimensions of both types of elements. An external magnetic field of 250 Oe was applied perpendicularly to the long side of the elements to create transverse uniaxial magnetic anisotropy, with the easy magnetization axis parallel to the short side in the plane of the stripe.

The magnetoimpedance effect of multilayered elements of both types was measured using an automatic system based on an Agilent HP E 4991A (Agilent Technologies, Santa Clara, CA, USA) impedance analyzer at room temperature for bare MI elements and in the presence of polymer composites filled with magnetic nanoparticles. Measurements were performed in a microstrip line, and electrical contact between the line and the film elements was established using liquid-metal InGa, providing high conductivity. An external magnetic field generated by Helmholtz coils was applied along the long side of the samples (longitudinal magnetoimpedance configuration) for the intensity of the driving current (I_ac_) to be about 1 mA. The frequency range of the alternating current passing through the element ranged from 1 MHz to 400 MHz.

The MI effect was described by the magnetoimpedance ratio (MI ratio) (1), the sensitivity of the MI ratio with respect to the external magnetic field (2), and the MI response Δ(ΔZ/Z) corresponding to the presence of filled polymer composite (3), which were determined as follows [16,28,29]:

Δ(ΔZ/Z) = 100 × (Z(H) − Z(H_max_))/Z(H_max_) (1)

S(ΔZ/Z) = δ(ΔZ/Z)/δH(2)

Δ(ΔZ/Z) = ΔZ/Z_control_ − ΔZ/Z_position_ (3)

where H_max_ = 100 Oe, δ(∆Z/Z) is the change in the MI ratio of the MI element with a change in the magnetic field, δH = 0.1 Oe, ∆Z/Z_control_ is the MI ratio of the MI thin film element itself, and ∆Z/Z_position_ is the MI ratio of the film element at a certain position of the magnetic filled composite sample. To analyze the frequency dependence of the MI ratio, the ∆Z/Z_max_ parameter was used as the maximum value of the MI ratio for the field dependence ∆Z/Z(H) at different frequencies.

The MI response was detected in a configuration useful for bioapplications, namely, for test experiments simulating the passage of a thrombus through a blood vessel. Samples of epoxy resin doped with magnetic particles were chosen as objects simulating a thrombus in a coronary vessel; their stray magnetic fields were detected using an MI element. The magnetic insert was positioned approximately 1.1 ± 0.2 mm above the element’s surface and could be moved perpendicularly to its long side. The position of the magnetic insert’s center relative to the film element along the OX axis varied within a step of ±1 mm (Figure 2c). This is a configuration that, in practice, can be realized not only for sensor system calibration, but also either manually for qualitative screening or using the scanning system for quantitative evaluation.

## 3. Results and Discussion

### 3.1. Structural and Magnetic Parameters of MNPs for Magnetic Fillers

Magnetic filler materials (Fe, Fe/Gd and Fe/Gd/Al_2_O_3_) were obtained on the basis of large batches of Fe (400 g), Al_2_O_3_ (300 g) and Gd (100 g), making their comparative analysis possible and precise. Although the amount of the materials after mechanosynthesis was limited by the total mass of the components in the initial state (12 g), due to the capacity of the particular ball-milling device, the process is scalable and can provide larger single batches, as requested for the application of nanodrugs [17], with means on the order of the size of the batches of initial materials. As is mentioned before, the initial set of materials consisted of spherical EEW MNPs of Fe, Al, and rapidly quenched Gd ribbons, mechanically cut into small pieces of a few millimeters long.

**Figure 3:**
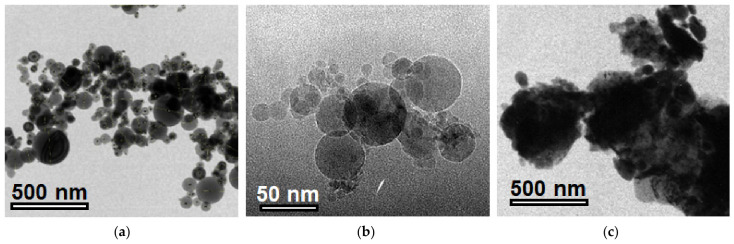
EEW MNPs of iron (**a**) and alumina (**b**); sediment MNPs of Fe/Gd (30 wt.% Gd) after complete ball-milling procedure (see also the main text) (**c**).

Figure 3 shows select TEM data for the initial states of Fe and Al_2_O_3_ MNPs and materials after ball-milling treatments. As is mentioned above, two fractions were formed: stable suspension of MNPs and sediment. For the epoxy composites, MNPs from suspensions were used.

Fe and Al_2_O_3_ MNPs in the initial state have shapes close to spherical (Figure 3). Fe MNPs had their iron oxide shell (of the order of 5 nm) formed during the fabrication process by the oxygen addition in order to passivate the surface. In the case of the iron MNPs, the major crystalline phase (about 96%) was face-centered cubic α-Fe, with lattice parameter a = 2.863 (±0.003) Å and the size of the coherent diffraction domains being 77 ± 3 nm. Two minor phases (γ-Fe, body-centered cubic with lattice parameter a = 3.592 (2) Å, and Fe_3_O_4_ magnetite with lattice parameter a = 8.360 (6) Å) corresponded to the shell of the MNP. The specific surface area of the Fe MNPs was 7.2 m^2^/g. XRD studies showed that Al_2_O_3_ particles were present in a single δ-Al_2_O_3_ phase material with lattice parameter a = 7.932 (±0.003) Å and coherent diffraction domains of 29 ± 3 nm. The specific surface area of the Al_2_O_3_ MNPs was 30.2 m^2^/g.

Measurements of the specific surface (S_sp_) value are widely employed in physical chemistry as an additional way to characterize powders, as they allow the simple calculation of the weighted-average diameter <d> using the following equation:

<d> = 6/(ρS_sp_) (4)

where ρ is the density of the material under consideration. The direct calculation of the weighted-average diameter <d> for MNPs in the initial state gives the numbers <d> = 106 for Fe MNPs and <d> = 50 nm for Al_2_O_3_ MNPs. However, strictly speaking, this equation contains two sources of the experimental errors that critically affect the weighted-average diameter calculation for the ensemble of MNPs: a sizeable deviation of the average shape from sphericity and ensembles of nanoparticles for which the density value cannot be determined with sufficient accuracy. This is why the advanced characterization of nanomaterials usually proceed from the need to determine the same parameter using several experimental methods [17].

Density, when applied to nanomaterials, is a very difficult parameter to determine, so, when calculating Expression (4), the bulk density of the material is most often used. The problem with nanomaterials, such as nanoparticles, is that the material at their surface differs significantly from the material in the bulk. For example, earlier [24], we discussed the properties of iron oxide nanoparticle inevitably having core–shell structure with a natural shell thickness on the order of 2 nm: for the MNPs with an average diameter of about 9 nm, the surface shell would occupy about 75% of the total volume. Therefore, even in the case of a single-phase nanomaterial, the accurate determination of its density becomes problematic, and the assessment of the error in determining the weighted-average diameter using Expression (4) becomes problematic.

The task becomes even more complex when attempting to create complex assemblies of nanoparticles, where changes in the size, shape, and phase composition of the components are possible. This is especially true for the magnetochemistry with two or more initial components. One such technique is ball-milling. This method was extensively studied for different materials that initially consisted of milli- or micro-sized components [30,31]. However, the field of the ball-milling processing of nanoparticles has very limited understanding. In addition, in the case when the study is focused on the mechanosynthesis of a stabilized suspension of magnetic particles, either electrostatically or sterically, the presence of these additional components also comes into play. The average diameter may be affected due to its component’s deposition onto the surface of MNPs. Phase composition can be changed during the ball-milling, and additional difficulties can appear for quantitative XRD analysis in multiphase cases, as, in conventional XRD, MNPs should be dried from suspension in the presence of a stabilizer.

The main purpose of synthesis in these studies was to fabricate stable suspensions starting from EEW α-Fe MNPs, Gd flakes, and EEW δ-Al_2_O_3_ MNPs. After special tests (not discussed here), we elaborated a protocol for ball-milling (in ethylic alcohol for 24 h, followed by partial drying to a paste-like state and additional processing in a ball-mill in an aqueous solution of 0.2% sodium citrate for 10 h), which resulted in the formation of two fractions in all cases under consideration: a stable suspension of MNPs and sediment. Stable suspensions of MNPs were the subjects of our investigation. They were stable (no visible sediments were detected) for at least the time period of 2 months. The first test of the stability of suspension was a definition of a degree of colloidal stability via the measurement of the Z-potential (Table 1)—very high values of Z-potential confirm the successful mechanosynthesis of electrostatically stabilized colloidal products. The number- and volume-averaged mean diameters (d_n_ and d_w_ respectively) were measured by standard protocol [24,25].

The results of the specific surface value are also given in Table 1. First of all, one can see that the specific surface area for the α-Fe MNPs (7.2 m^2^/g) and the product of the α-Fe mechanochemistry (13.6 m^2^/g) shows a sizable increase in S_sp_, almost twice as much, while the average size of MNPs decreases. However, the values for <d>, calculated on the basis of the initial mass of components, includes the knowledge of the phase composition and the density, i.e., a parameter that is quite difficult to define accurately at the nanoscale, even for pure elements. The results of the specific surface value are also given in Table 1.

**Figure 4:**
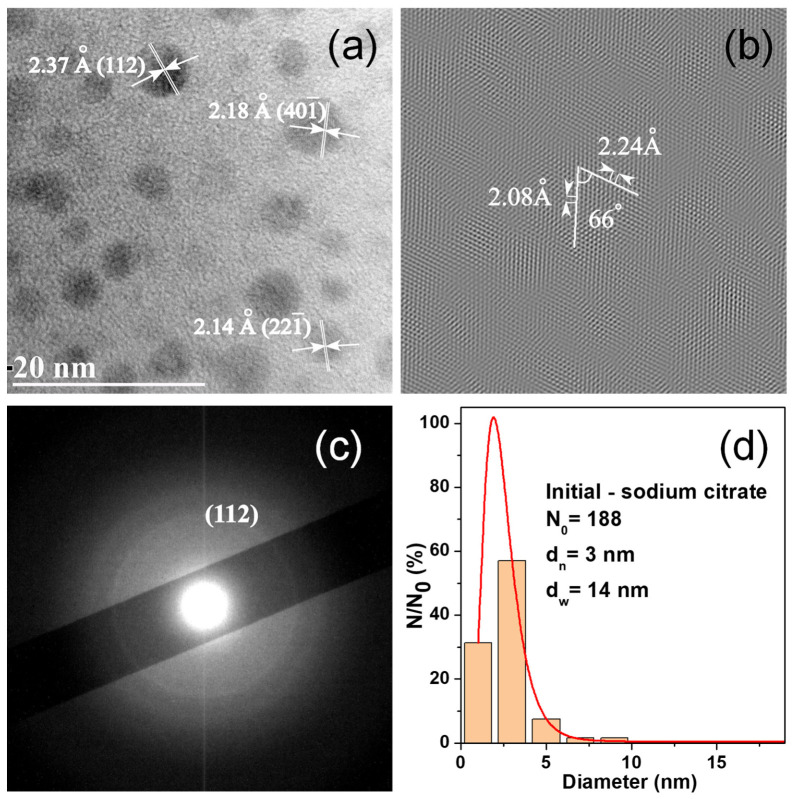
TEM images of spherical crystalline nanoparticles. The measured interplanar spacing and corresponding set of crystal planes of the Na_2_CO_3_ phase are indicated for select particles (**a**). High-resolution Fourier-filtered image of arbitrarily selected particle in a sample. The “[130]” crystallographic zone axis identified as Na_2_CO_3_ is visible (**b**). Electron pattern of a selected region from a sodium citrate sample. The main ring reflection of the sodium carbonate phase is visible—(112); interplanar spacing is 2.37 Å (**c**). Particle size distribution corresponding to (**a**) total number of particles N_0_; lognormal fitting for particle size distribution is also shown by a red line (**d**).

MNPs of dried suspensions were analyzed using TEM and DSL techniques. The presence of a dried aqueous solution of 0.2% sodium citrate was affecting the process of the calculation of the MNPs’ average sizes. Therefore, TEM studies were done for the separately dried aqueous solution of 0.2% sodium citrate without MNPs (Figure 4).

**Figure 5:**
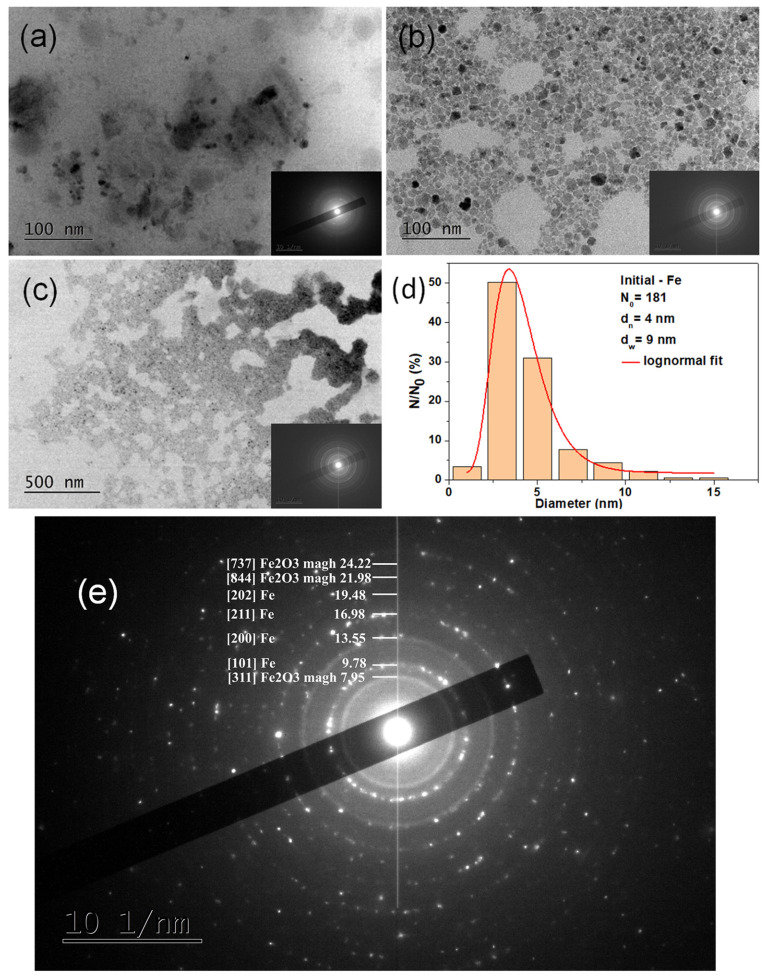
Representative images of Fe-based material after mechanosynthesis (**a**–**c**); insets show the microdiffraction patterns for corresponding area. MNP particle size distribution calculated on the basis of TEM data; lognormal fitting is also shown by a red line (**d**). Example of the microdiffraction parameter analysis with corresponding reflections and interplanar distances (**e**).

A sodium citrate solution was deposited on carbon-coated grids. High-resolution images revealed the crystallographic orientations of the monoclinic sodium carbonate phase and the corresponding interplanar spacings (Figure 5). Electron diffraction images reveal, in addition to an amorphous halo, the main reflection of this phase—(112)—corresponding to sodium carbonate, with the lattice parameter a = 2.37 Å.

**Figure 6:**
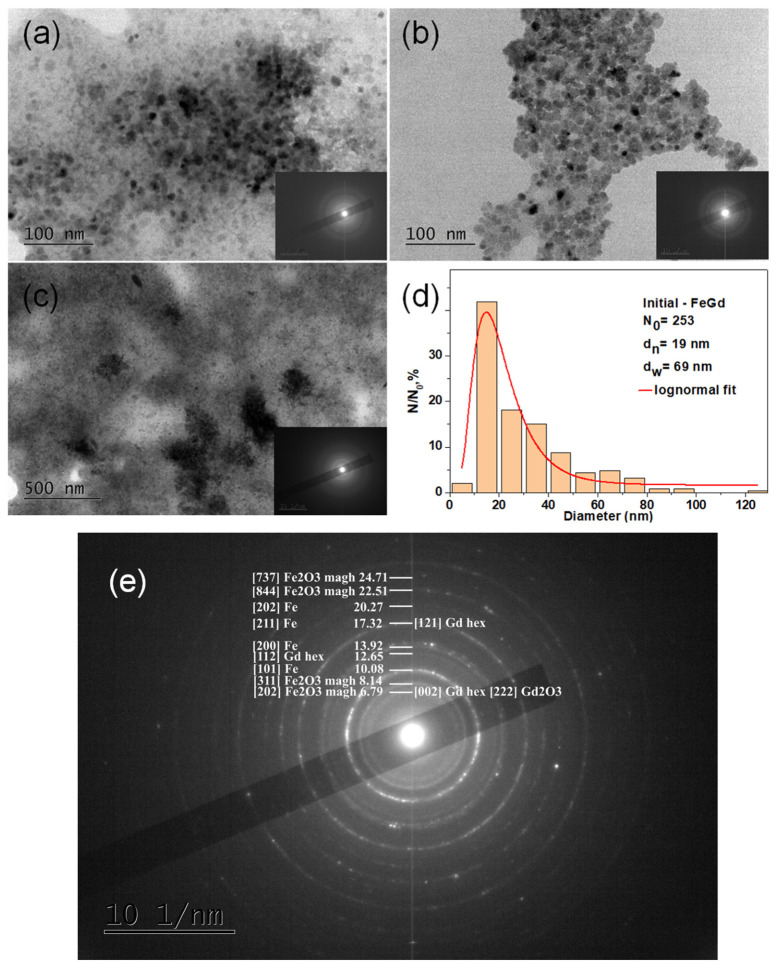
Representative images of Fe-/Gd-based material after mechanosynthesis (**a**–**c**); insets show the microdiffraction patterns for corresponding area. MNP particle size distribution calculated on the basis of TEM data; distribution is given for d_n_, and the value of d_w_ is given in the legend for comparison (**d**). Example of the microdiffraction parameter analysis with corresponding reflections and interplanar distances (**e**).

**Figure 7:**
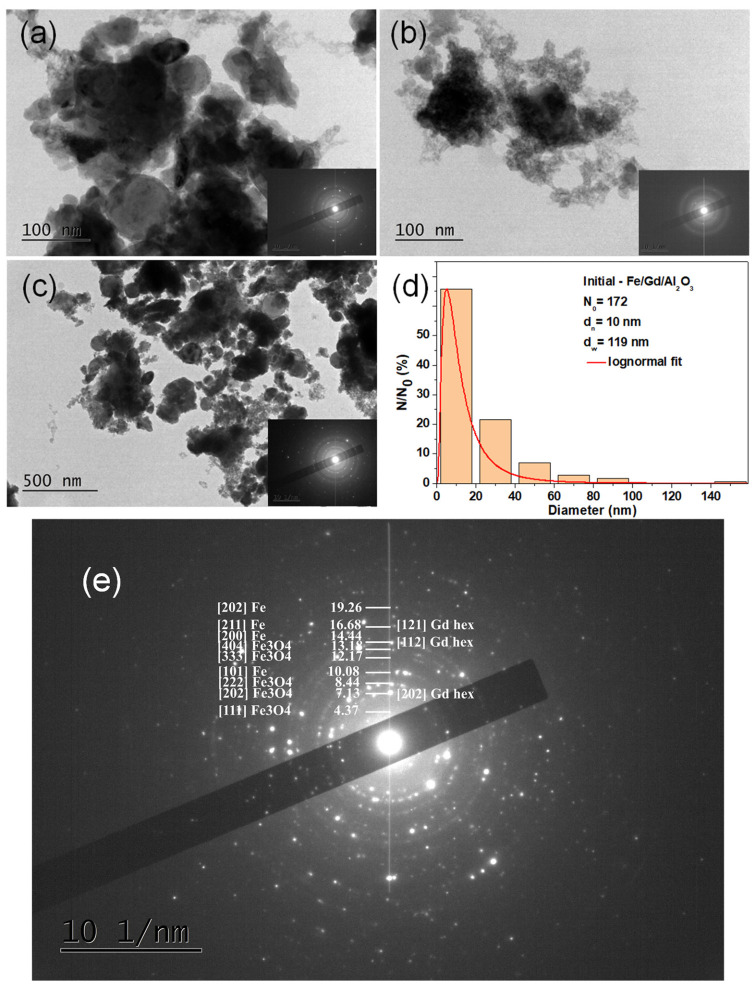
Representative images of Fe-/Gd-/Al_2_O_3_-based material after machanosynthesis (**a**–**c**); insets show the microdiffraction patterns for corresponding area. MNP distribution calculated on the basis of TEM data; distribution is given for dn, and the value of dw is given in the legend for comparison (**d**). Example of the microdiffraction parameter analysis with corresponding reflections and interplanar distances (**e**).

Sodium citrate exists as a mixture of hydrogenated phases that transition from one to another during dehydrogenation [32,33,34]. Decomposition occurs at temperatures above 300 °C, easily achievable under the electron beam of a TEM microscope. This means that the products collected from dry MNPs after mechanical synthesis may contain rather complex additions in the shape of non-metallic nanoparticles, which will certainly affect the specific surface measurement data, and they are difficult to identify in the highly statistical TEM studies. Figure 4d shows the particle size distribution for the products of a dried suspension of sodium citrate consisting of rather small particles (d_n_ = 3 nm). The existence of this additional fraction should move all experimentally defined distributions (for Fe, Fe/Gd, Fe/Gd/Al_2_O_3_ products) toward the smaller sizes. Figure 5, Figure 6 and Figure 7 show the results of TEM studies for solid deposits obtained for all three types of stable suspensions. Although the most representative images were selected, the goal was also to indicate possible differences within the same batch and to show materials at different magnifications.

One can see (Figure 5) that the stable suspension of Fe-based products is rather homogeneous over the large observation area, and the average size of the particles is small and similar to the average size of the particles of the dried sodium citrate. Their size distribution is well-adjusted by the lognormal function. However, for the particles of small size, it is not easy to separate the sodium citrate Fe MNP products. 

At the same time, all suspensions were obtained by mechanosynthesis using a water solution of sodium citrate. We suggested that the presence of sodium citrate would affect the accuracy of particle size distribution analysis in the same way. Therefore, as a first approximation, for the evaluation of the relative changes in the MNPs’ characteristics, one can neglect the presence of sodium citrate-related phases. Particles identified as Fe-based products of mechanosynthesis are close to being spherical in shape. In addition, according to the microdiffraction TEM analysis, this was confirmed by the calculations of the interplanar distances (Figure 5e). The ensemble of MNPs is mainly composed of spherical iron nanoparticles covered by a thin iron oxide (the maghemite phase) layer a few nanometers thick, but pure iron oxide MNPs are also present.

Figure 6 shows the results of TEM studies of Fe-/Gd-based dried suspension material. The stable suspension-related fraction is also homogeneous over the large observation area, but the average size of the particles was significantly increased with respect to the average size of the particles of Fe-based deposits after mechanosynthesis with sodium citrate. The particle size distribution is also well-adjusted by the lognormal function. Particles are close to being spherical. Here, it is possible to observe the existence of a small number of large particles (with diameter above 100 nm).One additional observation might be interesting. Figure 6c reveals the appearance of large loose accumulations of separate nanoparticles tending to form spherical clusters of about 200–300 nm in diameter. This kind of behavior—tendency to form loose clusters—was not observed for the case of Fe-based processed material. As a preliminary hypothesis, we can suggest that the mehanosynthesis of Fe-/Gd-based suspensions includes not only electrostatic stabilization by sodium citrate but also steric stabilization by the polymers that are the product of mechanosynthesis. 

However, further research steps are necessary to validate this idea. When milling the original gadolinium-containing powder, the TEM shows that the sample becomes somewhat homogenized—the reflections are more uniform, with a smaller proportion of point reflections. The intensity of the main iron line being “[101]” slightly increases visually, which may indicate the reduction of iron by gadolinium. Hexagonal gadolinium lines appear to partially overlap the iron lines. Under the conditions of these observations, the presence of gadolinium oxide is also possible.

Figure 7 shows the results of TEM studies for Fe-/Gd-/Al_2_O_3_-based dried suspension material. In this case, the result is very different from the two previous results. The material is much less homogeneous, and nanoparticles are represented by units with large shape and size variations. Often (Figure 7b), large particles contain a loose environment with long branches formed by smaller particles, up to the formation of defected networks at distances comparable with about 3 sizes of the central particle, showing the existence of complex self-organization processes.

Although it is hard to make a precise analysis, the general appearance and low contrast of the branches suggest that they contain only a small amount of metallic or oxide phases. The average size of the particles in the Fe-/Gd-/Al_2_O_3_-based dried suspension is significantly increased with respect to the average size of the particles in Fe-based and Fe-/Gd-based materials. The particle size distribution is reasonably adjusted by the lognormal function. Here, it is possible to observe the existence of small numbers of large particles (with caliper sizes in the range 100–150 nm). Here, we observe a large difference between the number- and volume-averaged mean diameters. The definitions of d_n_ and d_w_ for the particles with strong deviations from sphericity become unclear.

Microdiffraction analysis indicates that the ball-milling of iron and gadolinium powders in the presence of alumina abrasive results in the fragmentation of the diffraction pattern, with gadolinium aggregation and possible recrystallization. Such changes also complicate quantitative assessments due to the complex intensity distribution in the image. Iron oxide lines are detected, directly indicating a partial reduction of iron oxide, from oxidation state III to oxidation states II-III. An important point to be mentioned is the absence of the metallic alloyed phases of Fe, Gd or Al.

**Figure 8:**
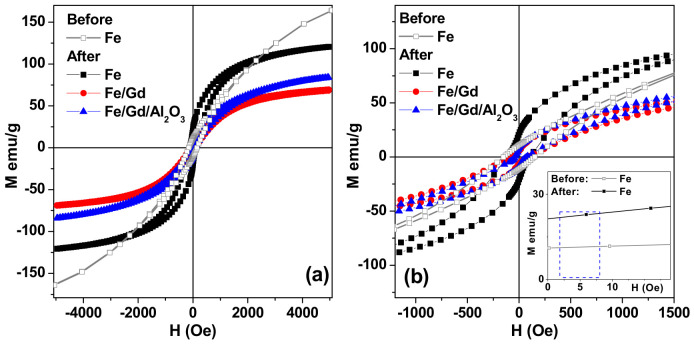
Magnetic hysteresis loops for α-Fe EEW nanoparticles in initial state (named “before” in the legend) and M(H) loops for all mechanosynthesis products (named “before” in the legend): (**a**) in the region of high magnetic fields; (**b**) in the region of low magnetic fields, where the inset shows M(H) branches for Fe powders before and after mechanochemistry in the decreasing field starting from magnetic saturation, and the dashed blue rectangle indicates the external field range most appropriate for magnetic biodetection.

To summarize the structural part, the Fe-/Gd-/Al_2_O_3_-based material was investigated under the supposition that Al_2_O_3_ is usually employed as an abrasive, i.e., a material that can help to decrease the average size of large flakes (gadolinium and large iron particles, in our particular case). The role of Al_2_O_3_ was to improve the stage of mechanosynthesis. The effect of additions of very small amounts of alumina was complex from the point of view of particle size distribution. For Fe-/Gd-based material, after mechanosynthesis, d_n_ = 19 nm and d_w_ = 69 nm, and, for Fe/Gd/Al_2_O_3_, d_n_ = 10 nm and d_w_ = 119 nm, i.e., in the Fe-/Gd-/Al_2_O_3_-based material, despite the lower d_n_ value, large particles were making a sizeable contribution. These structural peculiarities were reflected in the magnetic properties of polymer composites. A further understanding of the physical properties of the materials comes from an analysis of magnetic measurements performed at room temperature (Figure 8).

For the sake of convenience (to adjust the magnetic field intervals for magnetic and magnetoimpedance measurements with the prototype MI sensor), the value of magnetization in the external field H = 5000 Oe was assigned the M_s_ (saturation magnetization) value. The value of M(H = 5000 Oe) = 164 emu/g for α-Fe MNPs in the initial state is quite consistent with previous reference data [22] and the average size of MNPs covered by the iron oxide protective layer. It is worth mentioning the fact that the shape of the magnetic hysteresis loop of the α-Fe MNPs in the initial state is typical for reasonably uniform magnetic material with no inflection points and sharp changes in slope. In contrast, all M(H) hysteresis loops of the materials after mechanochemistry have no inflection points, and sharp changes in the slope of M(H) hysteresis loops most probably indicate the existence of different magnetic contributions. One of the contributions could be the sodium citrate itself. However, it is a non-magnetic (diamagnetic) salt, and therefore one cannot expect a sizeable change in M_s_ due to its or its products’ presence. Gadolinium and gadolinium oxides are paramagnetic near room temperature and also cannot significantly contribute to the M_s_ values of the samples.

M_s_ values for “α-Fe” samples before and after mechanochemistry were 164 and 122 emu/g respectively. Such a decrease is quite understandable, as the average size of the particles was decreased as a result of the treatment. This tendency was confirmed by the structural methods. The decrease in the saturation magnetization of the iron nanoparticle core and the oxide shell is usually calculated under the assumption that the specific saturation magnetization of the powder is additively composed of the ferromagnetic and ferrimagnetic contributions [22,25]. At the same time, the mechanonochemistry of α-Fe MNPs may increase the amount of iron oxide phases represented by small MNPs without a metallic core. Ms values for “α-Fe” samples before and after mechanochemistry were 164 and 122 emu/g respectively. 

Magnetic properties of Fe/Gd and Fe/Gd/Al_2_O_3_ were rather similar. M_s_ values of Fe/Gd and Fe/Gd/Al_2_O_3_ after mechanochemistry were 70 and 84 emu/g respectively. The decay to these numbers from 122 emu/g (for pure α-Fe initial material) was first of all due to 30 wt.% Gd in the mixture for mechanochemistry. Slightly higher Ms for Fe/Gd/Al_2_O_3_ can be explained if one takes into account the existence of the large MNPs in the particle size distribution, as large particles have higher magnetization at the nanoscale. The coercivity H_c_ (Figure 8) was the highest (about 170 Oe) for α-Fe samples in the initial state and about 50 for all samples after mechanochemistry, which does not contradict existing concepts about the non-monotonic dependence of the coefficient of iron nanoparticles in the nanoscale region.

It is especially necessary to mention the importance of the successful magnetic label detection of such parameters as remnant magnetization, e.g., M(H = 0 Oe), reached after saturation. For Fe samples after mechanochemistry, it is higher in comparison with M(H = 0 Oe) before mechanochemistry being 21 and 11 emu/g accordingly. The inset in Figure 8b shows that this ratio (approximately 2:1) is preserved over the field interval of 2 to 8 Oe, which is the most important for stray field detection.

### 3.2. Thermal Analysis of Curing of Magnetic Composites

Taking into account the structure and magnetic properties of magnetic powders after mechanochemistry, the following materials differ from each other in structure, but exhibit the magnetic properties most adequate for synthesizing the calibration samples for magnetic biodetection. The denominations of the epoxy composite samples are given in Table 2.

**Figure 9:**
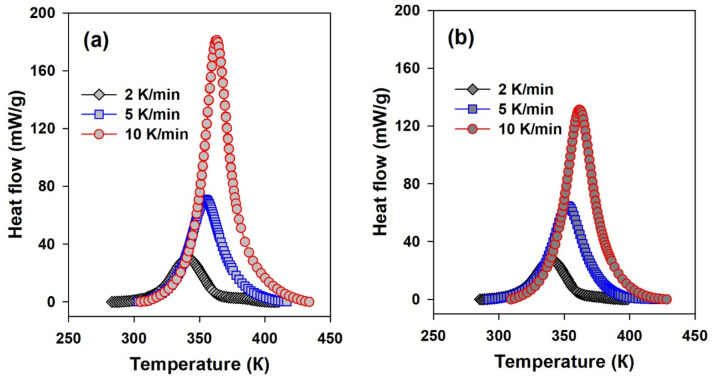
DSC thermograms of curing for epoxy composites at heating rates of 2, 5, 10 K/min. (**a**)—ED-20; (**b**)—ED-20 + 10% Fe/Gd/Al_2_O_3_ powders.

Figure 9 presents experimental thermograms of polythermal curing for ED-20 epoxy resin (Figure 9a) and for epoxy composite with 10% Fe/Gd/Al_2_O_3_ powders (Figure 9b) at three heating rates. Similar thermograms were obtained for all composites under study. The ordinate of the plot gives the heat flow in milliwatts for 1 g of composite in the calorimetric cell. All thermograms show distinct exothermal peaks, which correspond to the curing of composite. 

With the increase in the heating rate, the position of the peak maximum shifts to high temperatures, the peak height enlarges, and its shape becomes more symmetrical. The comparison of Figure 9a,b gives that the total evolution of heat, which is related to the peak height decrease with the addition of MNPs at any heating rate. This is because the heat flow is related to 1 g of the composite and the content of the epoxy resin is lower if the composite is filled with MNPs.

The integration of heat flow peaks with respect to the baseline gave the total enthalpy of curing for composites. These values are given in Table 2 with respect to the unit mass (1 g) of ED-20 in composite. One can notice that, for all composites, the values of the enthalpy of curing at different heating rates are statistically equivalent as, in all the cases, the ranges of experimental errors are overlapping. Therefore, the values of the enthalpy of curing were averaged over the heating rates.

**Figure 10:**
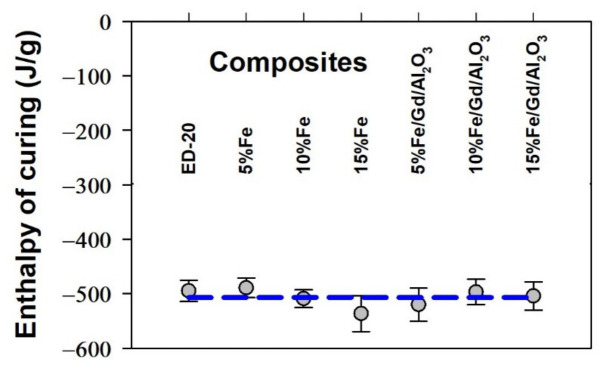
Enthalpy of curing of ED-20 epoxy in magnetic composites.

Figure 10 presents these average values of the enthalpy of curing for the magnetic composites under study. One can notice that, within the limits of the experimental accuracy, the values of the enthalpy of curing for all composites are statistically equivalent. This means that Fe or Fe/Gd MNPs did not influence the curing of the epoxy matrix of the composites. The average value of the enthalpy of curing over the whole number of composites was found—507 ± 25 J/g.

For the next step, let us see if MNPs influenced the kinetics of the curing of the composites. As is mentioned above, the increase in the heating rate resulted in the changes in the position and the shape of the exothermal peaks of curing (Figure 9). Based on this, the kinetic analysis of the curing process can be done using the conventional approach presented in the literature [35,36,37]. In general, the kinetics of the chemical reaction are given by the following equation [36,37]:

(5) $$\frac{d\alpha }{dt}=k\left(T\right)\times \;f\left(\alpha \right),\;$$

where *α* is the conversion of a reagent at a time *t*; *k*(*T*) is the kinetic constant of the reaction, which is dependent on temperature *T*; *f*(*α*) is the model function for the dependence of the conversion on the concentration of the reagent.

Irrespective of which type a model function *f*(*α*) might be, an activation energy of the reaction can be evaluated using experimental data, assuming that the kinetic constant obeys the Arrhenius equation for temperature dependence [36,37]:

(6) $$k\left(T\right)=Aexp\left(\frac{{-E}_{a}}{RT}\right),\;$$

where *A* is a constant; *E_a_* is activation energy, J/mmol; *R* is the gas constant, J/mol × K.

Equations (5) and (6) are then combined in Equation (7):

(7) $$\frac{d\alpha }{dt}=f\left(\alpha \right)\times Aexp\left(\frac{{-E}_{a}}{RT}\right),\;$$

The further transformation of Equation (3) is conventionally done using the Kissinger–Akahira–Sunose (KAS) method [38,39,40,41]. This gives the following logarithmic form of the KAS equation, which was elaborated for the evaluation of kinetic parameters in the polythermal mode of DSC:

(8) $$\ln\left(\frac{\beta }{T^{2}}\right)=\ln\left(\frac{AR}{g\left(\alpha \right)E_{a}}\right)-\frac{E_{a}}{RT},\;$$

where *g*(*α*) is the conversion function at a certain heating rate *β*, K/min.

To evaluate the activation energy (*E_a_*), the dependence of the conversion on temperature should be obtained at different heating rates *β*. Then, for the selected levels of conversion, the plots ln(*β*/*T*^2^) vs. 1/*T* are created. These plots are linearized, and the tangent gives the *E_a_*/*R* value for the selected conversion. Concerning a thermal process recorded by DSC, the conversion is conventionally calculated as the ratio of the partial integral under the part of the heat flow peak to the total integral under the peak as a whole. The partial integral is related to the specific temperature and is calculated as a part of the peak area up to this temperature.

**Figure 11:**
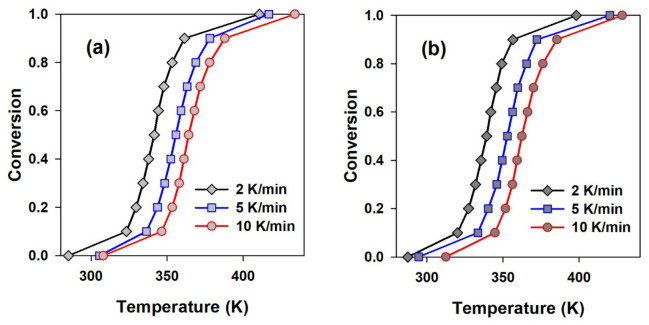
Conversion plots for the curing of epoxy composites at heating rates of 2, 5, 10 K/min. (**a**)—ED-20; (**b**)—ED-20+10% Fe/Gd/Al_2_O_3_.

Figure 11 presents examples of conversion plots for ED-20 epoxy resin and composite ED-20 + 10% Fe/Gd at three heating rates. Conversion plots were calculated based on thermograms in Figure 9. They are sigmoidal in shape and shift to high temperatures with the increase in the heating rate. The same plots were obtained for all magnetic composites under study.

**Figure 12:**
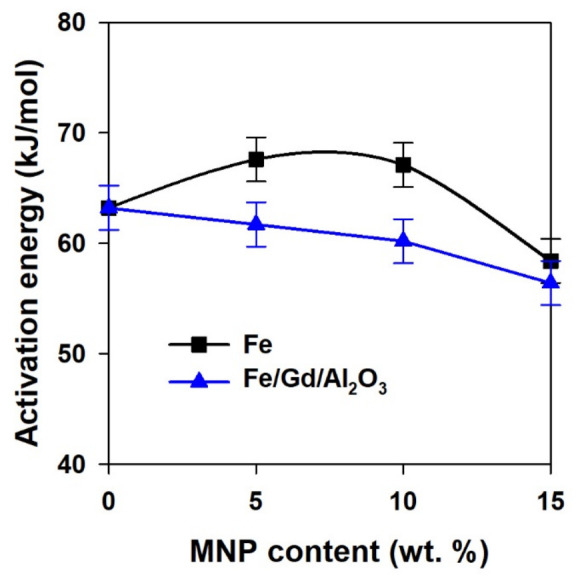
Dependence of the activation energy of ED-20 epoxy curing in magnetic composites on the content of Fe or Fe/Gd/Al_2_O_3_ MNPs.

Based on the conversion plots, like those given in Figure 11, the plots ln(*β*/*T*^2^) vs. 1/*T* were created, and the values of the activation energy of the curing of ED-20 epoxy in composites were calculated according to Equation (8). The values of the activation energy for conversions from 0.1 to 0.9 were very close and therefore they were averaged. Thus, the average value of the activation energy in the whole range of conversion for the curing of ED-20 epoxy was evaluated for the magnetic composites under study. As the curing was done in polythermal mode, this value corresponds to the whole temperature interval of the curing process. Figure 12 presents the dependence of the activation energy of curing on the content of MNPs in the composite. 

The values at the left ordinate axis correspond to the activation energy of curing for the individual epoxy resin ED-20. The found value of 63 ± 2 kJ/mol is in good agreement with the typical values reported in the literature [41]. Fe or Fe/Gd/Al_2_O_3_ show different influences on the activation energy of epoxy curing in the composite. In the case of Fe MNPs, there is a maximum in the plot, i.e., the addition of 5% or 10% Fe MNPs provides an increase in the activation energy and a retardation of the curing reaction, while 15% MNPs decreases the activation energy and promotes the reaction. The influence of Fe/Gd/Al_2_O_3_ MNPs is monotonous: across the whole range of MNP content, the activation energy diminishes and the curing reaction accelerates.

### 3.3. Static Magnetic Properties of Magnetic Composites

**Figure 13:**
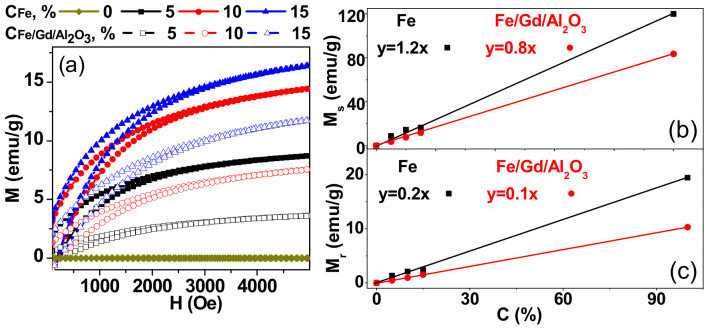
Field dependences of specific magnetic moments of composites with different concentrations of Fe- and Fe-/Gd-/Al_2_O_3_-related powders; percentages indicate the weight concentration of particles in the composites (0% epoxy resin without particles) (**a**). Concentration dependences of specific magnetic moment of composites based on magnetic particles: magnetic moment of technical saturation (in a field H = 5000 Oe) (**b**); remnant magnetization (**c**). For (**b**,**c**), the parameters of linear concentration dependences are also indicated.

The obtained magnetic composites (Table 2) were measured by VSM at room temperature (Figure 13) for the definition of their basic magnetic parameters.

Focusing on magnetic biosensors, one should take into account that compositions above 15% would never appear in real biological samples. Usually, they are below 5 wt%. For example, earlier, we have demonstrated the possibility of iron oxide nanoparticle detection for the samples mimicking natural tissue—synthetic ferrogels in the linear range of concentrations from 0.0 to 2.2 wt.% [42]. The sensitivity of the MI element is sufficient for ferrogel sample detection, even with 0.6 wt% maghemite nanoparticles. However, ferrogel sample measurement is a difficult task, as this mass is changing very quickly. A more time-stable composite is necessary, and the combinations proposed here are good candidates.

As to expect, the magnetic moment of technical saturation and remnant magnetization of the composites show a linear dependence on their weight concentration. The reasons for special attention to the composites with small concentrations of MNPs are the following. First of all, biological tissues and cells are not able to accumulate large amounts of the nanoparticles due to their toxicity and morphological incompatibility. In addition, in the majority of cases (usually up to 10 wt.%), filled composites’ properties can be described in the frame of the models of non-interacting MNPs [43,44,45,46]. Magnetometry is an accurate well-developed technique for measurements of small samples of magnetic composites. Usually, sample mass is limited by the appropriate size (a few millimeters of each dimension) and the magnetic moment range typically being above 10^−5^ emu. However, biomedical applications demand a non-destructive and non-contact evaluation of the concentration of magnetic nanoparticles in the particular zone (point of the therapy). Here, we propose to examine two types of small magnetic field detectors described in the Section 2. Both types of sensitive elements were fabricated using the sputtering technique [47,48,49,50].

### 3.4. Magnetoimpedance Multilayers and MI Measurements with Magnetic Composites

The first one (non-patterned, NP) is a classic symmetric MI multilayer for the detection of very small magnetic fields [40,48]. In the second case (square-patterned, SqP), the patterning of the top multilayer was provided during the deposition process through Cu-mesh. First of all, SqP structures were theoretically predicted to be more sensitive to the external magnetic field [28] in certain conditions.

**Figure 14:**
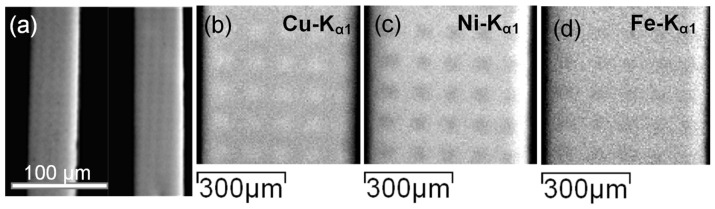
Surface geometry of the asymmetric [FeNi/Cu]_5_/Cu/[Cu/P{FeNi]_5_} MI elements observed by optical microscopy in two elements of the same batch (**a**). EDX analysis spectra of scanning electron microscope made separately for Cu-K_α1_ (**b**), Ni-K_α1_ (**c**) and Fe-K_α1_ (**d**) tests of patterned surface.

Figure 14 demonstrates the possibility of the fabrication of SqP structures using even a very simple technique involving metallic masks. Although the high-quality geometric contrast is difficult to achieve, even using scanning electron microscopy, due to large geometrical differences in the dimensions of the structural elements (thickness of 500 nm and length of about 50 microns), elemental analysis allows the accurate determination of the geometric parameters of patterned elements. For example, white squares with a shortage of Cu (Figure 14b) are complementary images for dark squares (Figure 14c,d), confirming that FeNi layers are situated on top of the central Cu-lead. Calculation based on ideal geometry (Figure 2b) shows that the active surface area of the SqP element is at least 3% higher in comparison with the classic symmetric element. However, due to shade effects of mask deposition, this value might be even higher.

**Figure 15:**
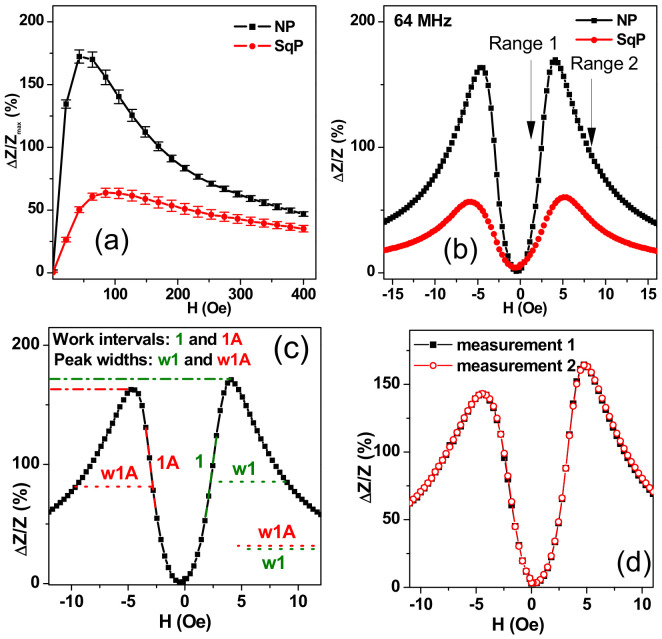
Frequency dependences of maximum MI ratio (error bars are included) (**a**) and field dependences of MI ratio for total impedance (**b**) for NP- and SqP-sensitive elements. MI curve of NP element at f = 64 MHz with indication of two linear ΔZ/Z(H) intervals (1 and 1A in the positive and negative field ranges, accordingly); w1 and w1A are the ΔZ/Z(H) peaks width measured at the half height of the peak in the positive and negative field ranges, accordingly. Measurement done starting from magnetic saturation in the high positive magnetic field (**c**). Two independent measurements (measurement 1 and measurement 2) using NP element and ED-20 + 15%Fe composite sample at the same position at 4.0 mm mounted independently (see also Figure 2) (**d**).

Both Fe- and Fe-/Gd-/Al_2_O_3_-based composites were measured using the protocol adapted for possible biodetection (Figure 2c). As the first step in MI prototype calibrations, the frequency dependence of the ΔZ/Z_max_(f) was measured for NP- and SqP-sensitive elements. Figure 15a shows typical thin-film MI multilayer dependences of ΔZ/Z_max_(f), with maxima close to 64 MHz. For a comparison of the performance of the MI structures, the driving current frequency f = 64 MHz was selected as the frequency for which the highest values of the ΔZ/Z_max_ ratio were obtained for both structures. This also means that, for f = 64 MHz, the highest sensitivity (ΔZ/Z/ΔH is a change in the MI ratio per unit of the external magnetic field) was achieved. Figure 15c demonstrates the level of repeatability of the MI measurements in the most difficult case: with filled polymer composite displaced to the ox point of 4.0 mm for two independent positionings of the composite in the same space point.

The NP element shows a much higher MI effect in comparison with the SqP element across the whole frequency range under consideration. Figure 15a shows external magnetic field dependences of total impedance ratio for the selected frequency of 64 MHz (maximum of ΔZ/Z_max_(f) value). The maximum ΔZ/Z ratio for NP element was about 160 and for SqP about 60%. A careful calculation of the linear ΔZ/Z(H) ranges gives the following numbers for the working interval of MI-sensitive elements (Table 3).

Usually, designers of small magnetic field detectors are interested in obtaining devices with their work point (middle point of the work interval) in low magnetic fields in the range 2–3 Oe. The case of the detection of the stray field of magnetic labels can be different. For magnetic label detection, two different magnetic materials work in one device and have field intervals where the materials demonstrate the highest performance. Their field interval may have (or not have) the intersection. In other words, the optimum operating conditions for different materials in one device may not correspond to the optimum conditions for their operation separately. We therefore provide analysis for both low- and high-field-linearity regions (range 1 and range 2, accordingly). One can notice (Figure 15b) that the MI curve of the NP element is not exactly symmetric with respect to the vertical axis. Here, we do not mean the small displacement with respect to H = 0 (below 0.3 Oe) caused by the impossibility to compensate the terrestrial magnetic field completely, but rather the observed ratio ΔZ/Z_max_(H < 0) ≠ ΔZ/Z_max_(H > 0). That is, the MI peak in the positive filled range (after saturation in the positive maximum experimentally available field) is slightly higher and narrower in comparison with the MI peak appearing in negative field after saturation in the positive maximum experimentally available field; here, w1 is the width of the peak for H > 0 and w1A is the width of the peak for H < 0. This asymmetry for the MI materials (without sources of the stray fields nearby) was observed many times and discussed in the scientific literature as asymmetric MI [51,52,53,54].

There are different approaches and models, and their detailed discussion is not possible within the scope of this article. However, we would like to mention one of the examples [54] related to the calculation of the transverse magnetic susceptibility, showing that the observed behavior is related to the very high sensitivity of the magnetic system to the circumferential magnetic field, induced by the ac current, close to the spin-reorientation phase transitions taking place in the magnetic film due to the magnetic anisotropy of a higher-order contribution. A model calculation explains that the peaks can differ in their shape: the MI curve has two peaks due to two orientation phase transitions. The first peak after magnetic saturation in the positive field (situated in the positive field) shows a jump in the susceptibility value because the transition at the positive field is of the first order. The second peak (situated in the negative field) is broader; it shows no special features, reflecting the fact that this transition is of the second order. Again, the magnetic anisotropy of a high order, which is not simply uniaxial magnetic anisotropy, playing an important role.

Coming back to Figure 15c,d, one can pay attention to the fact that measurements in the presence of filled magnetic composites decrease the ΔZ/Z_max_ value (for the same conditions), which is not surprising and essentially the method for the formation of the effective magnetic field of complex configuration. The understanding of the experimental observation, that the difference between ΔZ/Z_max_(H < 0) and ΔZ/Z_max_(H > 0) becomes slightly larger, can be ascribed to the development of the model based on the Landau–Lifshitz equation of motion for susceptibility calculations similar to the one in Ref. [54]. However, due to a certain distribution of the stray fields of individual MPs of the composite, the uniformity of the external field becomes additionally disturbed and the magnetization of the composite becomes less uniform, causing the difference in the condition corresponding to ΔZ/Z_max_(H < 0) and ΔZ/Z_max_(H > 0) in the presence of the composite. This part of the study requires further refinement.

**Figure 16:**
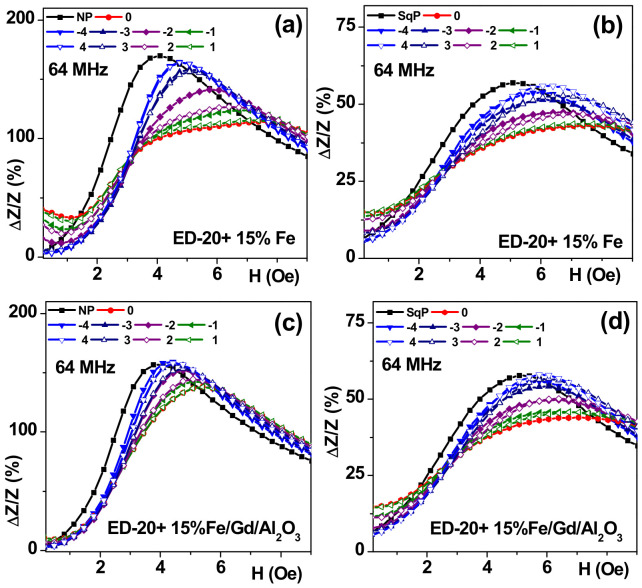
Field dependence of the MI ratio for magnetic composite detection with NP (**a**,**c**) and SqP MI element (**b**,**d**) for frequency f = 64 MHz. Numbers indicate the position of the magnetic composite in millimeters (see also Figure 2c). Parts (**a**,**b**) describe results for ED-20+ 15%Fe and parts (**c**,**d**) for ED-20 + 15%Fe/Gd/Al_2_O_3_ composites.

Figure 16 shows results of the magnetic detection of the composite sample with 15% magnetic filler of Fe or Fe/Gd/Al_2_O_3_ type. As the first step, the epoxy composite was magnetized in the high field (as is shown in Figure 2c), aiming to take advantage of non-zero remnant magnetization M_r_ for the creation of the stray fields of MNPs. MI responses were measured without composite followed by the measurement with composite at nine different positions (see also Figure 2c). One can see that the maximum of MI curves is observed in the field H = 4.1 Oe for NP and H = 5.1 Oe for SqP MI elements. As the center of magnetic composite approaches the central point of the sensitive element, the MI ratio curves shift toward higher fields, and the maximum value (peak of the curves) decreases, which is consistent with the results of previous MI studies [29]. Let us analyze Figure 16a as an example. The black curve corresponds to the MI response of the element without the presence of polymer composite. In this case, the MI response depends on the value of the external magnetic field H only. In the presence of the composite sample situated under the MI element at the position when projection of the center of the composite cylinder onto the MI element coincides with center of MI element (see Figure 2c), the MI response becomes most disturbed (Figure 16a, red curve). In a simple way, one can approximate the stray field of the composite to be a field of the dipole. The intensity of the stray field depends on the distance as 1/x (for large distances). Therefore, the larger the displacement x (Figure 2c), the smaller the contribution of the stray fields and the smaller the deviation from the MI response of the sensitive element in the absence of the composite. More details, including a particular example of calculation using finite-element modeling, can be found elsewhere [29].

For the MI ratio curves measured in the presence of composites with Fe particles, a similar trend is expressed to a greater extent, which is associated with a higher magnetic moment (higher H_r_) compared to Fe/Gd/Al_2_O_3_ particles. The MI response of the elements to different positions of the composites was calculated at a fixed frequency of 64 MHz in a field of 3 Oe, corresponding to a number approaching the upper limit of the working range of each element.

**Figure 17:**
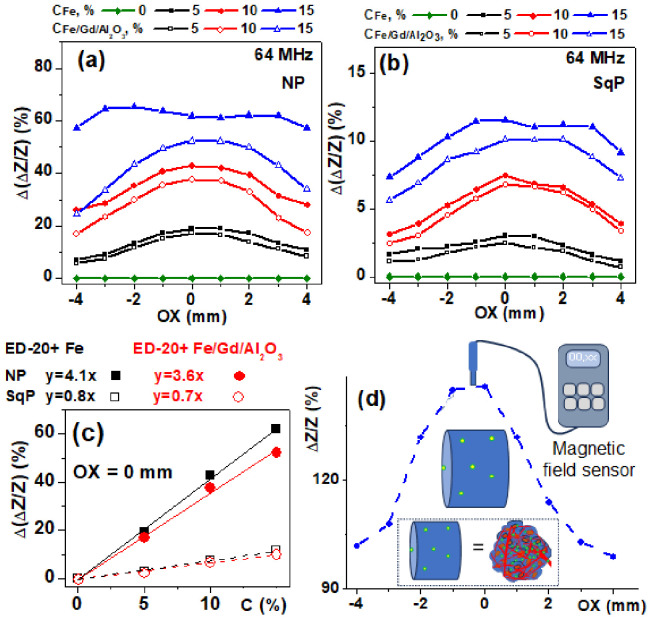
MI responses of NP (**a**) and SqP (**b**) elements at different positions of filled composites with different concentrations of magnetic particles. Dependence of the MI response on the particle concentration in the composite at its position of 0 mm relative to the MI element. Numbers for linear dependences Δ(ΔZ/Z) = kC are also indicated (**c**). MI response of NP element with ED-20 + 15% Fe composite for the magnetic field H = 4 Oe (see Figure 16a), showing the possibility of magnetic detection of stray fields of NPs using simple scan process and magnetic field MI sensor. Inset illustrates the concept of equivalence of stray fields of NPs embedded in polymer and tumor tissue from the point of view of biodetection (see also Figure 1c) (**d**).

The maximum MI response corresponds to the NP element (Figure 16 and Figure 17). 

Focusing on magnetic biosensors, one should take into account that compositions above 15% would never appear in real biological samples. Usually, they are below 5 wt%. Our goal was to analyze the properties of two different magnetic materials: multilayered MI-sensitive element and polymer composite filled with magnetic particles. The MI sensor’s important characteristics are sensitivity with respect to applied field and work interval. The latter is an external field interval for which MI response shows linear dependence on the applied field. The central part of the work interval is the work point. For each frequency of the driving current, work interval should be defined separately. However, the polymer composite’s properties are also dependent on the value of the external magnetic field, and the goal of designer of the device is to select materials and indicate conditions for which the proposed device would satisfy the request.

In magnetic field sensors, designed for the detection of stray fields of magnetic nanoparticles embedded into polymer matrix, the stray fields’ intensity at each particular value of the external field depends on the magnetic moment of the composite sample: the higher the concentration of the MPs, the higher the stray fields’ intensity. The effective field at the surface of the MI element becomes a sum of the applied external field and the stray fields of the composite sample. If the stray field of the composite becomes too strong, the effective field governing the MI response appears to be pulled out of the work interval, as happens for the 15% NPs composite. In this case, a comparison of the results for small concentrations and large concentrations becomes meaningless. This is just a demonstration that the proposed MI-sensitive element has a concentration limit that can be detected. For VSM measurements, this is not a problem.

The profiled SqP element is free from this drawback due to its lower sensitivity. However, at lower concentrations of magnetic particles in the composites, it exhibits a smaller MI response (Figure 17b). At a fixed central position OX = 0 mm, the MI responses depend linearly on the concentration of magnetic particles of both types (Figure 17c). The MI response of elements to composites with Fe/Gd/Al_2_O_3_ particles is slightly lower than the response to composites with Fe particles, which is associated with their lower magnetic moment (Figure 17c). Even so, it demonstrates the clear capability to detect the inclusion of the magnetic filled polymer composite in a weakly magnetic environment, satisfying the goal of magnetic biodetection.

The presence of gadolinium-based components opens the possibility of the development of multifunctional devices with different imaging approaches. Figure 17c shows the MI response of NP elements with the ED-20 + 15% Fe composite for the magnetic field H= 4 Oe (data taken from Figure 16a). One can clearly appreciate the possibility to detect the position of the magnetic filled composite, which, in practice, combined with a scanning system, can be used for tumor or stenosis detection. It is important to mention similarity between the Figure 13b,c and Figure 17c concentration dependences for the magnetic filler obtained by very different techniques, confirming the possibility to use MI sensors after appropriate calibration or measurements of basic magnetic characteristics.

Figure 17d shows the MI response of NP elements with the ED-20 + 15% Fe composite for the magnetic field H = 4 Oe (data taken from Figure 16a), confirming the possibility of magnetic composite detection using a simple scan process and magnetic field MI sensor. Here, the magnetic composite is represented as a filled polymer cylinder with NNPs. However, from the point of view of stray field detection, the filled polymer composite can be equivalent to the biocomposite, such as the tumor with incorporated magnetic particles (generic representation of which is given in Figure 1c).

The substitution of part of the iron by gadolinium, as is mentioned above, may reduce biomaterial toxicity but add extra parameters, making the system multifunctional. First of all, MNPs having a magnetic moment and can be a part of magnetic field-assisted therapy, where an external magnetic field helps to deliver the magnetic carrier and associated drug to the point of care. It is also known that natural tissue has such properties as specific uptake and the retention of the nanoparticles [17]. This means that part of the nanodrug arriving to the point of therapy would not stay there for an unlimited amount of time. Therefore, additional systems for the control of the number of the particles (magnetic field sensor) could monitor the MNP concentration. Fe-/Gd- or Fe-/Gd-/Al_2_O_3_-based materials, apart from the carrier and marker of concentration roles, can be also used for imaging, ensuring the multifunctional use and appropriate biocompatibility.

A magnetic field sensor with sufficient sensitivity to detect stray fields of certain amounts of the nanoparticles of a particular type must be calibrated with the same nanoparticles for the possibility to measure their concentration in a small concentration range. If any kind of nanomaterial is expected to work as a part of a magnetic biosensor, it must be used for the calibration of the biosensor. Nanomedicine allows the drug to be from the same batch only. This means that initially the batch of nanoparticles must be sufficiently large for the synthesis of the stabilized suspension and composites for calibration.

We describe all steps of fabrication of MI biosensor from MNPs in a large batch sufficient for the synthesis of the stabilized suspension and composites for calibration. The thorough characterization of all materials might be useful for other researchers. This is also the experimental report on fabrication previously described theoretically for MI structures with a profiled top layer. In recent years, special efforts were made in search of an MI thin film and multilayered structures, with a focus on specialized applications [55,56]. This work attempts to further extend this area of research and applications.

We made an attempt to provide an advanced characterization of obtained materials using traditional physical chemistry techniques and magnetic measurements of different types. Although a complete understanding of the materials was not possible, the proposed methodology might be useful for other researchers working in the field of applied nanomaterials for biomedical applications.

## 4. Conclusions

In this work, ensembles of Fe and Al_2_O_3_ nanoparticles were fabricated by the electric explosion of wires. Gd ribbons were obtained by the rapid quenching technique. Three types of materials (Fe, Fe/Gd and Fe/Gd/Al_2_O_3_) were obtained by the ball-milling mechanosynthesis of Fe particles (100%), Fe and Gd particles (70 and 30% accordingly), and Fe (69 wt.%), Al_2_O_3_ (1 wt.%), and Gd (30 wt.%) flakes. Fe, Fe/Gd and Fe/Gd/Al_2_O_3_ fillers were obtained as a result of mechanosynthesis in the state of a very stable aqueous suspension with Z-potential of about −54 mV. Biomedical applications require the synthesis of large batches of stable aqueous suspensions. Fillers from suspensions were used for the synthesis of epoxy composites mimicking the natural tissue with embedded magnetic particles of the same type. Obtained materials from dried suspensions were used for the fabrication of filled composites with 0, 5, 10, and 15% content of MNPs in epoxy resin ED-20, i.e., corresponding to the concentration range of interest for biomedical applications.

Thin-film MI-sensitive elements of two types were prepared by the sputtering technique: conventional [FeNi(100 nm)/Cu(3 nm)]5/Cu(500 nm)/[Cu(3 nm)/FeNi(100 nm)]5 with symmetric geometry of top and bottom layers (NP), and asymmetric [FeNi(100 nm)/Cu(3 nm)]5/Cu(500 nm)/[Cu(3 nm)/P{FeNi(100 nm)]5} element with patterned top multilayered structure (SqP). They showed a maximum value of MI ratio about 160% for NP and about 60% for SqP.

Complex ensembles of magnetic nanoparticles obtained by electrophysical methods in combination with a ball-mill and polymer composites on their basis were subjected to a thorough characterization of their physical and chemical properties, including magnetic measurements.

MI measurements using sensitive elements of NP and SqP types revealed that the sensor prototype response was affected by the presence of filled magnetic composites in the shape of cylinders with a diameter of 5 mm and a height of 4 mm, showing the capacity of designed thin-film elements to detect the stray fields of model composites as a function of magnetic filler concentration and the position of the polymer sample. This result opens the possibility of the development of a new research line of applications of iron- and gadolinium-containing nanoparticles, which can be used for magnetic imaging and detection by magnetic field sensors, extending the functional properties of biomedical devices and therapies.

## Figures and Tables

**Table 1:** Z-potential, specific surface (S_sp_), weighted-average diameter (<d>) calculated for the initial component composition (before ball milling), and average diameters obtained by TEM (D_TEM_) and DSL (D_DLS_) measurements.

Fillers (Based on)	Z-Potential, mV	S_sp_, m^2^/g	<d>, nm	D_TEM_, nm	D_DLS_, nm
Fe	−54.4 ± 3.9	13.6	56	d_n_ = 4; d_w_ = 9	d_n_ = 32; d_w_ = 48
Fe/Gd	−56.8 ± 2.7	21.3	36	d_n_ = 19; d_w_ = 69	d_n_ = 26; d_w_ = 57
Fe/Gd/Al_2_O_3_	−56.8 ± 2.7	10.5	73	d_n_ = 10; d_w_ = 119	d_n_ = 57; d_w_ = 84

**Table 2:** The enthalpy of curing at three heating rates for epoxy composites filled with MNPs after mechanosynthesis.

Composite	Enthalpy of Curing of Epoxy (J/g)		
	2 K/min	5 K/min	10 K/min
ED-20	−492 ± 20	−516 ± 20	−477 ± 20
ED-20 + 5%Fe	−481 ± 18	−487 ± 18	−499 ± 18
ED-20 + 10%Fe	−527 ± 16	−505 ± 16	−495 ± 16
ED-20 + 15%Fe	−552 ± 33	−559 ± 33	−499 ± 33
ED-20 + 5%Fe/Gd/Al_2_O_3_	−522 ± 30	−550 ± 30	−489 ± 30
ED-20 + 10%Fe/Gd/Al_2_O_3_	−491 ± 24	−523 ± 24	−477 ± 24
ED-20 + 15%Fe/Gd/Al_2_O_3_	−495 ± 26	−53 3 ± 26	−484 ± 26

**Table 3:** Characteristics of different MI-sensitive elements calculated for the driving current frequency of 64 MHz.

Element’s Type	Linear Field Range 1, Oe	Sensitivity 1, %/Oe	Linear Field Range 2, Oe	Sensitivity 2, %/Oe	H for ΔZ/Z at f = 64 MHz, Oe
NP	1.20–3.30	125.0	5.0–7.4	61.0	4.1
SqP	0.75–3.80	14.3	5.8–8.7	7.8	5.1

## Data Availability

The original contributions presented in this study are included in the article. Further inquiries can be directed to the corresponding author.
